# The Relationship Between Insight and Anxiety Within a Diverse Alzheimer’s Disease Sample

**DOI:** 10.1002/alz.084853

**Published:** 2025-01-03

**Authors:** Jewel Heald, Marie Forgeard, Jason Osher

**Affiliations:** ^1^ William James College, Newton, MA USA

## Abstract

**Background:**

A growing number of individuals and families are affected by Alzheimer’s disease (AD) across the United States. Changes in insight and levels of anxiety have been extensively but independently investigated in this population. In contrast, research on the relationship between insight and anxiety has been limited and has yielded inconsistent results.

**Method:**

The present study used the NACC Uniform Dataset (*N* = 18,671) to examine cross‐sectional associations between insight and anxiety in a diverse AD sample in order to better understand their potential role in individuals’ presenting symptoms and overall distress.

**Result:**

Results showed that although higher insight was associated with higher anxiety, this effect disappeared when controlling for other variables. Instead, age of onset, disease severity, and depression severity emerged as significant predictors of greater anxiety. In addition, depressive symptoms also moderated the relationship between insight and anxiety. For participants with lower depression, high insight was associated with lower anxiety. Results also supported the notion that race/ethnicity may play a role in the relationship between insight and anxiety. Overall, Black/African American participants in this study had somewhat lower levels of anxiety than other participants. In addition, within participants with low insight only, Hispanic/Latino/a/x participants had higher levels of anxiety than others.

**Conclusion:**

These findings provide valuable insight into the emotional experiences of patients with AD and may help inform clinicians about potential treatment and intervention options.

